# Emotional, Behavioral, and Psychological Impact of the COVID-19 Pandemic

**DOI:** 10.3389/fpsyg.2020.566212

**Published:** 2020-10-02

**Authors:** Ana Luisa Pedrosa, Letícia Bitencourt, Ana Cláudia Fontoura Fróes, Maria Luíza Barreto Cazumbá, Ramon Gustavo Bernardino Campos, Stephanie Bruna Camilo Soares de Brito, Ana Cristina Simões e Silva

**Affiliations:** Interdisciplinary Laboratory of Medical Investigation, Faculty of Medicine, Federal University of Minas Gerais (UFMG), Belo Horizonte, Brazil

**Keywords:** SARS-CoV-2, COVID-19, behavior, psychological changes, social isolation, restricting measures, mental health

## Abstract

The emergence of SARS-CoV-2 in December 2019 prompted consternation in many parts of the world. Due to its fast dissemination, the World Health Organization declared a pandemic in March 2020. Aiming to contain the spread of the virus, leaders of many countries restrained social movement, targeting to flatten the curve of contamination with social distancing. This review aimed to analyze how human behavior has changed throughout this period. We also approached the key components of the emotional reaction to the pandemic, how internal and external factors, such as personality traits, gender, the media, the economy and the governmental response, influence the social perception of the pandemic and the psychological outcomes of the current scenario. Moreover, we explored in depth the groups at increased risk of suffering mental health burden secondary to these circumstances. These include the healthcare professionals, elderly individuals, children, college students, black subjects, latin and LGBTQ+ communities, economically disadvantaged groups, the homeless, prisoners, the rural population and psychiatric patients. We also discussed several measures that might minimize the emotional impact derived from this scenario. It is crucial that the health authorities, the government and the population articulate to assist the vulnerable groups and promote emotional and psychological support strategies. Moreover, it is fundamental that the population is provided with accurate information concerning the COVID-19 pandemic.

## Introduction

In December 2019, a cluster of pneumonia cases was reported in the province of Hubei, China (Lu et al., 2020). It was then discovered that the infection was caused by a virus, named SARS-CoV-2. Subsequently, the illness caused by this virus was termed Coronavirus disease 2019 (COVID-19). Data from the World Health Organization (WHO) Guidelines indicate that by January 14th 2020, 1 day after the first recorded case outside of China, only 41 cases were confirmed (World Health Organization [WHO], 2020b). At the present, statistics taken from the WHO Coronavirus Disease (COVID-19) Dashboard by September 5th announce 26.5 millions of confirmed cases worldwide, with over 871 thousand deaths (World Health Organization [WHO], 2020c).

Restricting measures have been implemented in several countries as an attempt to slow down the dissemination of the SARS-CoV-2. China (Wang C. et al., 2020), Italy (Briscese et al., 2020), and the United Kingdom (UK) (Holmes et al., 2020), for example, carried out strict “lockdown” regulations, while other countries, including the United States of America (US) (Imperial College of London, 2020) and Brazil (Simões e Silva et al., 2020), have delivered “stay home” recommendations. In many places, means of transportation were shut down, public spaces were closed and only essential services kept functioning; albeit with restrictions and preventive measures.

However, as the world authorities seem to focus on the infectious aspect of the pandemic, a rise has been observed in mental health disorders (Brooks et al., 2020; Holmes et al., 2020). Indeed, during this ongoing health crisis, those affected by emotional, behavioral and psychiatric disorders tend to be more numerous than those affected by COVID-19. As a matter of fact, the fear of contracting COVID-19 seems not to be as high as concerns about the psychological and social impact of the pandemic, as reported in a United Kingdom survey (Mental health Covid-19, 2020). Particular groups appear to be at higher risk for this kind of mental health impact, including frontline healthcare workers, the elderly, children, college students, the LGBTQ+ community, homeless individuals and those in economic vulnerability, rural community, foreigners and psychiatric patients (Holmes et al., 2020; Khan et al., 2020; Salerno et al., 2020; Wood et al., 2020). Indeed, the emotional stress linked to the current scenario may potentially aggravate previous psychiatric conditions or may precipitate its symptomatology (Yao et al., 2020). A critical aspect of this context is that, due to physical distancing, many elective appointments have been canceled and mental health support systems have been suspended, even though remote assistance is rapidly increasing (Holmes et al., 2020).

This review aims to discuss the impact of COVID-19 for the mental health of the overall world population, in addition to its causes and ramifications. The topics of greater relevance in the scientific literature so far have been included, most of which concern not only the healthcare professionals and authorities, but the entire population as well. Furthermore, some measures that ought to be taken to minimize the emotional burden of the pandemic were debated.

## Methods

Data were obtained independently by six authors, who carried out a comprehensive and non-systematic search in the PubMed, Cochrane, Scopus, SciELO, and Google Scholar databases. Search strategies included terms as: “COVID-19,” “SARS-CoV-2,” “anxiety,” “depression,” “psychiatric disorders,” “social isolation”, “behavior changes”, “psychiatric patients”, “mental health”, “suicide”, “media”, “racism”, “healthcare workers,” “elderly,” “domestic violence,” “sleep,” “LGBT community,” “homeless,” “foreigners,” “rural community,” “informal settlements.” The search was conducted between May 14th and May 26th. This article was subsequently updated between May 26th and September 5th. The search emphasized recent articles, published case series, consensus statements, guidelines, meta-analyses, systematic reviews and prospective cohort studies, critically reviewed and selected by the authors. Research has also been made in informative official website public domains and in the references contained in the previously data collected.

## Restricting Measures Due to the Pandemic

### Terminology on Quarantine, Social Isolation, and Social Distancing

In the context of COVID-19 pandemic, the terms “social distancing,” “social isolation,” and “quarantine” have been used mostly as synonyms in the media, in communication with the public and even in scientific papers (Brooks et al., 2020). However, there are great differences between these designations, even though there is not always an agreement on the terminology. “Quarantine” refers to extreme restrictions of movement of those exposed or potentially contaminated by the virus, in order to minimize the spread of the pathogen. Moreover, the term “quarantine” ought to be used in the context of groups or at community level (Dsouza et al., 2020; Sánchez-Villena and de La Fuente-Figuerola, 2020). “Social isolation” refers to the restriction of social movement of those infected with the disease (Dsouza et al., 2020; Sánchez-Villena and de La Fuente-Figuerola, 2020). Meanwhile, “social distancing” is a preventative measure recommended to the general population to flatten the curve of the contagious disease. In this scenario, people are advised to stay at home and use services as little as possible, as well as to avoid agglomerations, maintain the recommended distance of one meter from each other and take precautionary measures to avoid infection (Covid-19, 2020). Nevertheless, the use and the comprehension of these terms should not be so inflexible. In fact, the term “social isolation” has also been used to express the source of subjective feelings of solitude that may accompany the social distancing measures, especially for those who are already at enhanced risk of suffering from loneliness. Notwithstanding, the term “social disconnection” is used in this review to encompass this framework.

### Source of Psychological Impact Related to the Restricting Measures

It is undeniable that the restricting measures imposed to contain the COVID-19 pandemic have a severe impact on the mental health of the population. Nonetheless, it is yet unclear what promotes such negative effects. It is possible that these repercussions derive directly from the restrictive strategies and reduced social mobility (Bavel et al., 2020; Brooks et al., 2020; Pfefferbaum and North, 2020; Wang G. et al., 2020). However, the emotional and psychological outcomes of the pandemic may also be secondary to the intrinsic changes that the restricting measures cause in lifestyle habits and socioeconomic scenario (Brooks et al., 2020; Zhu et al., 2020).

## Key Components of the Emotional and Behavioral Response to COVID-19 Pandemic

The emotional and behavioral response to COVID-19 pandemic is multifactorial. It relies not only on external components, but on personal and innate ones as well. Nonetheless, the reaction to the current circumstances seem to have predominant elements in the overall population. A significant increase in feelings of functional impairment, boredom, stigma, worry, phobia, frustration and anger has been observed (Ahmadi and Ramezani, 2020; Brooks et al., 2020; Pfefferbaum and North, 2020; Restubog et al., 2020; Sher, 2020a; Teufel et al., 2020). In this topic, some selected factors must be discussed thoroughly due to its pivotal influence on the mental health impact of the pandemic.

### Fear and Uncertainty

Unlike other virus outbreaks of the 21st century, such as SARS and MERS, which were primarily disseminated in hospital environments (Bai et al., 2004; Cauchemez et al., 2016), COVID-19 is unique in the way that it has spread far beyond health centers’ borders. With the entire population at risk, the necessary restricting measures have created an unparalleled scenario, dominated by fear and uncertainty. Even though fear is an essential adaptive mechanism that humans and other species have developed to cope with threats in the environment, it can only be supportive for those who feel capable of dealing with the menaces presented specifically to them. To those who consider themselves as unable to handle such risks, fear can trigger defense responses (Bavel et al., 2020). And so, in a conjuncture where the fear is not only of death but also of the repercussions in a myriad of different spheres, including family organization, schools closure, social isolation and economic consequences, it is vital that close attention is paid to the mental health of the individuals (Ornell et al., 2020). In fact, previous studies showed that fear positively associates with depression, anxiety, perceived infectability and germ aversion (Ahorsu et al., 2020). Furthermore, another detrimental consequence of fear is the stigmatization and discrimination of those infected or exhibiting symptoms of COVID-19 (Ahorsu et al., 2020).

Although fear has several destructive outcomes, one of the most maleficent one is suicide. In the COVID-19 pandemic, there have been numerous reports of suicidal behavior due to fear-related issues, for instance, fear of being infected (Dsouza et al., 2020; Mamun and Ullah, 2020), fear of infecting others (Mamun and Griffiths, 2020), fear of being quarantined (Dsouza et al., 2020) and fear of the mental health impact (Sher, 2020b). A particular illustration of this is a Bangladeshi 40-year old woman who took her own life in a hospital bathroom after being refused medical care due to the staff’s fear of SARS-CoV-2 infection (Mamun et al., 2020a).

Finally, it must be observed that the adjustment to the new life of social distancing may differ according to age groups, gender and other variables surrounding the individuals. Therefore, given the importance of fear in the pandemic context, scales addressing this feeling have been developed and might be helpful to the comprehension and management of this emotional component (Ahorsu et al., 2020; Sakib et al., 2020).

### Stressors

In the pandemic background, stressors must also be considered in the assessment of the emotional and neuropsychological impact. These mainly include COVID-19-related circumstances, such as potential exposure to the virus and loss of loved ones, as well as secondary adversities due to economic difficulties, unavailability of food, psychosocial effects, disruption of future plans and underlying physical and psychological conditions (Islam et al., 2020; Pfefferbaum and North, 2020).

#### Economic Factors

The ongoing pandemic caused by COVID-19 has set off a distinguishable economic crisis in considerable domains of work and business, including manufacturing, retail, travel and trade (Restubog et al., 2020). Unemployment is in the rise and even the most stable and former professionals are having their work threatened. The International Labor Organization estimates that there will be 25 million new unemployed individuals by the end of the second quarter of 2020 (Restubog et al., 2020). In addition, surveys with US workers before and after previous economic downturns state that unemployment is not the only possible detrimental outcome, since pay cuts, reduction in work hours, increased work demand and challenging working conditions are possibly part of a contingency plan for this pandemic (Restubog et al., 2020). Financial loss has been profoundly linked to psychological distress and is considered a risk factor for mental health disorders, with long lasting effects. The disruption or even bankruptcy of business, unpaid debts, stress of losing job, poverty, inability to provide support to the family and food insecurity are only a few examples that portray the extremely harsh scenario regarding the financial impact secondary to this pandemic (Bhuiyan et al., 2020; Dsouza et al., 2020; Mamun and Ullah, 2020). Indeed, the lack of basic supplies, including water, food, clothes and accommodation, seems to be a particularly deleterious source of frustration, anxiety and anger (Brooks et al., 2020).

Additionally, a disturbing matter is that the economic impact represents one of the main risk factors for suicidal behavior (Conejero et al., 2020; Vandoros et al., 2019). During the pandemic, cases of suicide due to financial downturns have been reported in several countries, particularly in those experiencing more severe crises than developed countries, such as India (Dsouza et al., 2020; Griffiths and Mamun, 2020), Bangladesh (Bhuiyan et al., 2020; Griffiths and Mamun, 2020) and Pakistan (Mamun and Ullah, 2020). Using the data available in the International Labor Organization’s press release in March 2020, a study has estimated that, in the best-case scenario, the rise in unemployment rates will provoke an increase of about 2,135 suicides in a year worldwide (Kawohl and Nordt, 2020). Therefore, the number of individuals who might seek help from mental health services is expected to substantially increase in the context of the COVID-19 pandemic (Kawohl and Nordt, 2020).

#### Domestic Violence

As a matter of fact, as the “stay home” recommendations remain, it is crucial to remember that home is not always a safe place for everyone. It can also be a residence for distortion of power and abuse, which is supported by studies that suggest that forced proximity, along with economic stress and disaster-related instability, are risk factors for aggression and domestic violence (Bavel et al., 2020; Usher et al., 2020). Furthermore, distancing measures also represent, for those living in violent places, diminished access to community-based and familial support, with fewer opportunities to ask for help (Usher et al., 2020). Fear of COVID-19 and threats about contamination can even be used as a coercive mechanism to maintain the abuse. As a consequence, for example, those suffering from domestic violence may be less inclined to go to the hospital on account of fear of infection. Ultimately, the social distancing, albeit essential to contain COVID-19, may exacerbate the violence and maintain it less visible (Usher et al., 2020).

Indeed, in the United Kingdom, a domestic abuse organization reported that calls to its domestic violence helpline increased by 25% in the 7 days following the announcement of tighter social distancing and lockdown measures by the government (Bradbury-Jones and Isham, 2020). In Australia, some police departments reported a 5% increase in domestic violence-related calls, while Google announced a 75% growth in internet searches for domestic abuse support (Usher et al., 2020). Additionally, there was a 32–36% increase in domestic violence complaints in France and a 21–35% increase in the US after the implementation of the social distancing measures (Usher et al., 2020). This pattern is equivalent to what has already been observed in previous epidemics (Usher et al., 2020).

### Changes in Daily Habits

Analysis on sleep quality during the SARS-CoV-2 pandemic also indicated that there has been a rise in sleep disturbances, a critical condition associated with anxiety, depression, and suicidal behavior (Sher, 2020a). Furthermore, diminished sleep quality promotes short temperament and, as a consequence, complicates family cohabitation (Islam et al., 2020).

Another interesting inquiry was related to news monitoring: a study suggested that higher averages of time (≥ 3 h) spent focusing on the virus outbreak was positively correlated to the development of anxiety symptoms (Huang and Zhao, 2020), but also with social responsibility values and compliance to social distancing recommendations among US adolescents (Oosterhoff et al., 2020). Contrarily, less engagement in risk prevention behaviors was observed in individuals who were apparently prone to “optimism bias,” the belief that they are less likely to acquire the disease than others. This principle is also seen in other diseases, including lung cancer (Soofi et al., 2020).

Moreover, an Italian survey performed in April 2020 assessed the changes in eating and lifestyle habits of 3,533 individuals, aged between 12 and 86 years. It was observed that 34.4% of responders had increased appetite during this period, whereas 17.8% had less appetite. As a result, nearly half of the participants of the study perceived weight gain during the pandemic. Additionally, it was observed that although there were no differences in physical activity in the group of individuals who did not play any sports before the COVID-19 lockdown, the training frequency has increased amongst those who were physically active. Around 3% of smokers have quit smoking in this period, probably due to the fear concerning increased risk of respiratory distress and mortality from COVID-19 (Di Renzo et al., 2020).

### Individualized Response to Stress

In times of psychological distress, emotional reactivity is deeply influenced by individual differences and stress-mediated contexts. A study with the Italian general population aimed to observe the gender and personality traits that more substantially associated with psychological impact during the COVID-19 pandemic (Moccia et al., 2020). The results showed that individuals with anxious, cyclothymic and/or depressive temperaments are predicted to suffer greater emotional impact secondary to the current scenario. Meanwhile, male gender, as well as secure and avoidant adult attachment style were protective for the risk of higher psychological burden (Moccia et al., 2020).

Moreover, in a different line of research, a Chinese study proposed to understand the differential psychological distress among distinct populations affected by the pandemic. It was observed that individuals who had experienced SARS-CoV-2 infection had significantly increased prevalence of depressed mood, somatic symptoms and anxiety-like behavior (Zhang W.R. et al., 2020). Post-traumatic stress disorder (PTSD) was found to affect 96.2% of hospitalized patients with COVID-19, whereas depression was also higher in COVID-19 patients (Vindegaard and Benros, 2020). Furthermore, having an infected friend or family member has been associated with higher anxiety levels (Duan L. et al., 2020).

## Psychological Disorders Secondary to the COVID-19 Pandemic

The COVID-19 pandemic may intensify psychological disorders or precipitate others, for instance, anxiety, depression, PTSD, alcohol misuse, obsessive-compulsive behaviors, panic and paranoia (Dubey et al., 2020; Islam et al., 2020). A nationwide survey in China with over 52 thousand participants had almost 35% of the respondents experiencing psychological distress due to the SARS-CoV-2 (Qiu et al., 2020). In this study, women appeared to be more vulnerable to stress than men, although this result is not consistent in literature (Huang and Zhao, 2020). Therefore, some of the most cited psychological consequences of the pandemic will be further addressed.

### Anxiety and Depression

Anxiety, one of the main evaluated subjects, has been significantly increasing in society during this pandemic (Huang and Zhao, 2020; Li et al., 2020; Qiu et al., 2020; Teufel et al., 2020). A research group in China analyzed the online posts from about 18,000 Chinese social media users before and after the declaration of COVID-19 in China on January 20, 2020 and found an increase in words that mirror negative emotions including anxiety, depression, and anger (Li et al., 2020). One particular kind of anxiety is worth mentioning: health anxiety. It is characterized mainly by catastrophic misinterpretations of bodily sensations, dysfunctional beliefs about health and illness and maladaptive coping behaviors. Harmful consequences can derive from this condition, including excessive hand washing, social withdrawal, panic purchasing and overspending in resources such as hand sanitizers, medications and protective masks (Asmundson and Taylor, 2020b). In fact, especially for the suspected cases of COVID-19, the development of obsessive-compulsive symptoms may be a consequence of anxiety related to their health status (Dubey et al., 2020). The same rising tendency has been seen for depressive symptoms (Bavel et al., 2020; Pfefferbaum and North, 2020; Restubog et al., 2020; Sher, 2020a). Interestingly, groups with less education seem to be more susceptible to these manifestations in an epidemic context, especially due to unreliable access to information and apprehension to its academic formation (Pfefferbaum and North, 2020).

### Post-traumatic Stress Disorder

Another alarming condition that can be expected to increase is post-traumatic stress disorder (PTSD) (Brooks et al., 2020; Dutheil et al., 2020; Gunnell et al., 2020), similar or worse to what happened in previous epidemics, such as H1N1 Influenza and Ebola (Xu et al., 2011; Cénat et al., 2020). The adverse effects of this illness are not manifested immediately and mental health support must be prepared to deal with this issue in a few months. PTSD is more likely to take place after longer periods of social disconnection and it is associated with increased suicide risk by 2–5 times (Thibodeau et al., 2013; Brooks et al., 2020). PTSD patients are also less prone to seek help from authorities, possibly due to few available information about this subject, fear of stigmatization, beliefs that symptoms may disappear over time and concerns about the cost of mental healthcare (Dutheil et al., 2020).

### Alcohol Addiction

During the lockdown, some countries also prohibited alcohol sales. The arguments to sustain the restricting conditions included impaired ability of those under the influence of alcohol to implement the preventive measures, the influence of drinking in domestic violence, its impact to the immune system and, finally, the high cost of acute drinking for the emergency services (Nadkarni et al., 2020). Nevertheless, higher numbers of abstinence syndrome appeared as a consequence within patients who suffer from addiction (Narasimha et al., 2020). In a psychiatry emergency service in Bangalore, India, twice the number of severe abstinence syndrome (seizures, delirium tremens, and hallucinations) occurred per day after lockdown (Narasimha et al., 2020). Furthermore, rise of the black marketing of alcohol, consumption of non-consumable alcohol and even suicide in those suffering from addiction have been reported in India (Nadkarni et al., 2020). In fact, it has been suggested that alcohol consumption is an important risk factor for the decompensation of psychiatric disorders and, ultimately, may favor individuals to commit suicide, particularly the fragile and more vulnerable ones (Conejero et al., 2020).

Another complex consequence of such measures concerns those in recovery or wanting to recover from alcohol abuse. Since autonomy is crucial to sustain behavioral changes that result in the discontinuation of drinking, and since the patients have, during this period, restricted access to services such as Alcoholics Anonymous, prohibiting alcohol sales can be detrimental to recovery (Nadkarni et al., 2020). Additionally, the social distancing, the anxiety and the negative thinking that have been exacerbated in the pandemic situation may trigger relapse (Nadkarni et al., 2020). In fact, in other countries where alcohol sales were not prohibited, such as in the United Kingdom, the consumption of alcohol during lockdown significantly increased (Nadkarni et al., 2020). It is important, then, that countries prohibiting alcohol sales carefully address its impact for those who suffer from addiction.

## Mental Health Vulnerability

Several groups are more vulnerable to greater emotional, behavioral and psychological impact of the COVID-19 pandemic. The most cited ones will be addressed in this discussion. Nevertheless, other groups at increased risk for the mental health repercussions of the pandemic include those with pre-existing health conditions, those living in care homes, domestic caregivers and COVID-19 patients and their family members (Dubey et al., 2020; Khan et al., 2020).

### Healthcare Professionals

One of the main groups in this category is the one with the healthcare providers during the pandemic, specifically frontline workers. In the alarming context of this health emergency, these professionals are put through different circumstances and afflictions, which include fear of being infected and infecting others, higher workload, significant pressure, pain of losing patients and colleagues, the yet unpredictability nature of the virus, inadequate testing, limited treatment options and disruption of regular routine, along with insufficient personal protective equipment and other medical supplies, especially in developing countries (Chew et al., 2020; Lancet, 2020; Mamun et al., 2020c; Pfefferbaum and North, 2020). Evidence reports that such conditions might make them more vulnerable not only to physical symptoms, including headache and sore throat (Chew et al., 2020), but also to mental health burden, with an increase in rates of anxiety, depression, stress, irritability, insomnia, anger, and frustration (Brooks et al., 2020; El-Hage et al., 2020; Pfefferbaum and North, 2020; Zhang J. et al., 2020). Having an organic disease appeared as an independent risk factor for these outcomes in previous studies (Zhang J. et al., 2020). As an illustration, a study in China concluded that half of the frontline healthcare professionals had symptoms of depression and anxiety, 70% had psychological distress and many also reported insomnia (Mesa Vieira et al., 2020). Previous epidemics had a similar pattern, as 29% of the healthcare workers may have had emotional distress after the SARS epidemic in 2003 (Holmes et al., 2020). This group is also at risk for the development of PTSD (Dutheil et al., 2020).

Moreover, as the COVID-19 pandemic is currently at the center of the news broadcast, the public gains access to the scientific data practically at the same time that it becomes available to the medical community. Therefore, in the midst of fear and anxiety, there is great pressure on healthcare professionals to be constantly updated on the release of new studies, as well as to prescribe the experimental treatments for their patients, in spite of insufficient high-quality evidence (González-Padilla and Tortolero-Blanco, 2020).

The extent of mental health vulnerability seems to vary amongst different populations within the healthcare staff. It has been reported that young women are at higher risk for adverse psychological repercussions than men (El-Hage et al., 2020; Lai et al., 2020). Moreover, nurses are also more likely to be affected than physicians (El-Hage et al., 2020; Lai et al., 2020; Tsamakis et al., 2020). Interestingly, one study observed that non-frontline nurses were more prone to emotional impact than the frontline group, which seems to be due to their greater working experience and psychological preparation (Ghaffari et al., 2020). Although results from other researches evidenced the opposite (Lai et al., 2020), it brings attention to the importance of providing psychological preparation and assistance to healthcare professionals during the COVID-19 outbreak.

### Elderly

As part of an important risk group for COVID-19, the elderly are currently being instructed to remain at home and self-isolate (Armitage and Nellums, 2020). Nonetheless, it has been demonstrated that older adults are at higher risk for anxiety and depression when put in situations of social disconnection (Armitage and Nellums, 2020; Conejero et al., 2020). To those who do not have close family or friends and to those whose only social contact is out of the home, these can be particularly dawning times. Many individuals within this group rely solely on community centers, places of worship, voluntary work and social care, activities which have been severely restrained by the COVID-19 outbreak (Armitage and Nellums, 2020). In addition, many older individuals have smaller access and/or literacy to social networks, which prevents them from maintaining virtual connection with others (Mesa Vieira et al., 2020). Therefore, the psychological and emotional impact is tremendous. The social disconnection causes and aggravates loneliness, neglect, depression and anxiety, all of which can produce long-term health consequences (Banerjee, 2020; Bavel et al., 2020). Moreover, the context of this pandemic might increase suicide behavior amongst older adults. As an example, after the SARS epidemic in 2003, suicide rates among elderly individuals were increased by 30% (Holmes et al., 2020). The solitude in the elderly has also been suggested to be accompanied by biological modifications that make this group more vulnerable to commit suicide, which includes the elevation of inflammatory markers and expansion of peripheral blood mononuclear cells (Conejero et al., 2020). Furthermore, it has also been suggested that social disconnection may worsen neurodegenerative disorders, such as Alzheimer’s disease (Conejero et al., 2020; Plagg et al., 2020).

An additional topic of discussion that further elucidates the emotional impact of the pandemic among the elderly is the phenomenon of “ageism”. In the early phases of the COVID-19 outbreak, the disease had been predominantly portrayed as an illness that affects almost exclusively the older adults (Ayalon, 2020). At present, this stereotype has been proven erroneous, as age itself is not a reliable criterion to predict the health impact of SARS-CoV-2 infection (Ayalon, 2020). Notwithstanding, albeit the scientific evidence of such a statement, the social marginalization and segregation of the elderly persists throughout the population (Ayalon, 2020; Colenda et al., 2020). Indeed, in some countries, the gradual relaxation of the social distancing recommendations does not seem to apply to the older adults, who are consistently advised to self-isolate (Ayalon, 2020). Moreover, there is a general belief that the safety of this group should be sacrificed for the greater good of society, particularly in detriment to the economy (Ayalon, 2020; Colenda et al., 2020). In this scenario, the aggravation of the intergenerational tension can be observed in social media content. As an illustration, the offensive hashtag #boomerremover appeared in over 4,000 posts in Twitter in a 10-day period following the pandemic declaration of WHO in March (Jimenez-Sotomayor et al., 2020). In this framework, there is a destructive increase in the mental health burden of senior citizens, which must be urgently addressed.

### Children

Children, especially the young ones, are also in a position of vulnerability during the pandemic. This happens because, at home, they suffer with limited social connection, crucial for identity and well-being at young ages, reduced physical activity, loneliness and boredom (Fegert et al., 2020; Jiao et al., 2020; Loades et al., 2020), which may result in long-term effects. Indeed, the mental and physical health, as well as productivity in adult life, is deeply rooted in the childhood years (Loades et al., 2020; Wang G. et al., 2020). Data from previous epidemics demonstrate that children who experienced isolation measures were five times more prone to demand mental health services and more inclined to experience PTSD (Loades et al., 2020). It has also been demonstrated that children who are out of school (i.e., weekend and summer holidays) tend to have longer screen times, irregular sleep patterns and less favorable diets (Wang G. et al., 2020), which can be exceptionally harmful in longer periods of time such as the yet unknown duration of this pandemic. Furthermore, the economic recession, the restricting measures and the overall family stress may be accompanied by an increase in domestic violence and child maltreatment, situations that impact the mental health of children (Fegert et al., 2020). Adolescents with previous mental health disorders require particular attention since disruption of school routine can decline their mental health status (Khan et al., 2020). Moreover, the current events have further prompted the expansion of remote work, whereas schools and daycare centers had to interrupt their activities. In this setting, family and work environment have merged and decreased performance can be seen in both spheres, as stress intensifies (Mental health Covid-19, 2020).

Following the distancing measures, social media has become an important resource to maintain social interaction. Even though its use might alleviate some of the mental health impact of the isolation, it is essential to analyze its negative impact in children and adolescents (Deslandes and Coutinho, 2020; Ni et al., 2020). First, consuming indiscriminate information about the pandemic may trigger stress, anxiety, panic and depression. This effect is even more intense in younger individuals that do not have the discernment to filter information (Deslandes and Coutinho, 2020). Second, the excessive use of the Internet might create an addiction, compromising the development of a healthier routine during the pandemic, which is also composed by study, leisure, and exercise activities (Deslandes and Coutinho, 2020). Third, digital social networks are extremely based in the virtual construction of a self-image and visibility, which, especially for the youngest, might mediate self-esteem through the pursuit of social approval. Simultaneously, social media can be a violent place. As a consequence, its excessive use may contribute to self-harm actions through virtual challenges, in which the participant has assignments related to self-mutilation and even suicide that should be filmed and posted. The online search for the term “challenges online” has increased since the implementation of the restricting measures (Deslandes and Coutinho, 2020). Ultimately, elevated Internet use is associated with behavioral problems such as neglecting personal life, relationship disorders, mood dysfunction and sleep disturbances, as well as increased anxiety and depression levels during the pandemic (Duan L. et al., 2020).

### College Students

Since universities have temporarily closed during this world health emergency, college students are also vulnerable to major changes in their routine and, as a consequence, to the psychological impact of the pandemic (Khan et al., 2020). As a matter of fact, having the graduation affected by the pandemic has already been significantly associated with increased depression rates (Duan L. et al., 2020). Factors that may aggravate this situation include living away from family, instability of family income and insufficient access to technology in order to attend online classes (Cao et al., 2020; Khan et al., 2020). In fact, the mental health impact of online classes is a topic that deserves further evaluation, since it might lead to overburden (Dubey et al., 2020). As an extreme example, a suicide pact related to online classes has been reported between a private university student and his mother. Similarly, suicides due to depression after an exam postponement and due to inability to access online classes have also been announced (Mamun et al., 2020b).

### LGBTQ+ Community

LGBTQ+ individuals, in general, have worse mental health and well-being compared to non-LGBTQ+ peers, especially for the persons of color (Fish et al., 2020; Salerno et al., 2020). As a consequence, during the COVID-19 pandemic, they face particular stressors that can trigger deleterious psychological outcomes (Fish et al., 2020; Phillips et al., 2020; Salerno et al., 2020).

For the LGBTQ+ youth, for instance, the social distancing measures might lead to confinement in unsupportive homes, increasing their exposure to discrimination, violence and rejection from their family (Fish et al., 2020). Previous researches have demonstrated that one third of the LGBTQ+ youth undergo family rejection and that these individuals are six times more prone to depression and eight times more prone to suicide (Salerno et al., 2020). Simultaneously, the LGBTQ+ youth, during the pandemic, experience less access to essential social connections, identity-based alliances and school-based mental health services (Fish et al., 2020; Salerno et al., 2020). As a result, this group is more vulnerable to anxiety, depression, suicide behavior, PTSD, substance abuse and self-harm (Fish et al., 2020; Salerno et al., 2020). For this reason, online communities have emerged as important support resources for this group (Fish et al., 2020).

Moreover, the LGBTQ+ elders are twice as likely to live alone, four times less prone to have children and more inclined to be segregated from their family (Salerno et al., 2020). Therefore, social distancing measures may exacerbate loneliness and previous mental health conditions (Salerno et al., 2020). Finally, the levels of poverty, lack of health-insurance and unemployment are higher amongst LGBTQ+ individuals, which aggravate the impact of the pandemic in their mental health (Phillips et al., 2020; Salerno et al., 2020).

### Black and Latin Communities

Structural racism imposes unequal access to healthcare and protective resources among different racial and ethnic populations. For instance, many individuals of color do not have adequate housing, a main social determinant of health. Additionally, nearly one third of the black Africans and one fourth of Black Caribbean in the United Kingdom are workers in essential services without the possibility of working from home (Farquharson and Thornton, 2020; Liu and Modir, 2020). Furthermore, there is an insufficient amount of well-resourced hospitals in many primarily black and latin communities (Liu and Modir, 2020). As a result, the COVID-19 pandemic has an increased impact on the individuals of color, which may lead to enhanced fear of infection and worse mental health outcomes (Farquharson and Thornton, 2020; Liu and Modir, 2020). In fact, black Americans have the highest COVID-19 mortality rate among the racial groups in the United States and early data reported that 33% of all deaths were of black people, even though this group composes 13% of the United States population (Liu and Modir, 2020).

Furthermore, in the course of the pandemic, the stigmatization of racial minorities exacerbates, causing rejection, social disconnection and physical violence (Farquharson and Thornton, 2020). As an example, the early recommendation to wear face masks in public resulted, for the black community, in increased racial profiling and police violence (Liu and Modir, 2020). Therefore, the narrative “we’re all in this together” regarding the COVID-19 outbreak has been proven inaccurate, and the communities of color are a vulnerable group for the mental health repercussions of the pandemic (Farquharson and Thornton, 2020; Liu and Modir, 2020).

### Foreigners

The continuous threat of SARS-CoV-2 has aggravated ethnic prejudice and intolerance toward stigmatized groups, especially toward Chinese people (Asmundson and Taylor, 2020a; Bavel et al., 2020). This is not unprecedented in History: Jewish people were linked to the Black Death, HIV was believed to be disseminated by the LGBTQ+ community and the western African population was discriminated against during the Ebola outbreak (Coates, 2020). In this pandemic, it is likely that the novelty of the virus and the uncertainty surrounding its potential outcomes in several spheres of society have triggered fear and anxiety that endorse the xenophobia behavior (Asmundson and Taylor, 2020a). There have been reports of discrimination within social and political contexts: Chinese restaurants having to shut down due to reduced number of costumers (Asmundson and Taylor, 2020a), Chinese individuals being barred from entering certain establishments and even the United States President referring to COVID-19 as the “Chinese virus” (Devakumar et al., 2020). Moreover, immigrants have decreased access to healthcare services, adequate housing and clean water, especially if undocumented (Liu and Modir, 2020; Mesa Vieira et al., 2020). To manage such reality, multidisciplinary measures are necessary to correctly inform the population on public health risks, to notify discriminatory acts and to support those affected by harmful misconceptions (Rzymski and Nowicki, 2020).

### Individuals in Economic Vulnerability

As in natural hazards, the economically disadvantaged people seem to be more susceptible to the threat and more likely to be minced by it (Bavel et al., 2020). For instance, a study has shown that groups with lower income may have been performing less physical exercises during this period, due to less access to Internet and technological tools. This is evidence that, besides the economic impairment, these individuals are also more vulnerable to physical and psychological repercussions of social isolation (Peçanha et al., 2020). Furthermore, there has been correlation between socioeconomic deprivation and ability to adopt preventive measures, enhancing the risk inequality (Atchison et al., 2020).

Those living in informal settlements have particular stressors that decline their mental health. For instance, space constraints, violence and overcrowding implicates in decreased capability to adhere to the social distancing measures and, as a consequence, in increased fear of contamination (Corburn et al., 2020). Furthermore, basic needs such as access to water, waste collection, sewers and adequate housing may not be available. Moreover, most of the individuals living in slums are informal workers and, therefore, are more vulnerable to the economical impact of the pandemic (Corburn et al., 2020). Finally, racism, xenophobia and stigmatization of the poor have raised during the pandemic, which implicates in even worse outcomes regarding the mental health of these populations (Corburn et al., 2020).

### Homeless Individuals

Homeless individuals compose another vulnerable group for contracting COVID-19 and for the psychological impact of the pandemic (Hsu et al., 2020; Khan et al., 2020; Mesa Vieira et al., 2020). First, this group faces difficulties in taking preventive measures for COVID-19, such as hand washing and self-isolation. Conversely, they are more prone to risky behaviors such as substance abuse and the sharing of needles (Wood et al., 2020). Second, many individuals have an increased prevalence of comorbidities and chronic diseases compared with people of similar age, including mental health disorders like bipolar disorder and schizophrenia (Khan et al., 2020; Mesa Vieira et al., 2020; Wood et al., 2020). Third, they face enhanced obstacles to receive treatment for previous medical conditions, especially considering their reduced accessibility to telehealth services (Wood et al., 2020).

### Prisoners

The vulnerability of the prison population in the COVID-19 pandemic does not seem to be thoroughly explored by the scientific community (Hewson et al., 2020). Notwithstanding, this group of individuals comprise numerous risk factors for worse mental health outcomes secondary to the current circumstances. This magnified emotional impact is a result of several aspects. The frequency of pre-existing psychological disorders, neurodevelopmental health, substance misuse, suicide and self-harm is already increased in this group compared to the rest of the population (Hewson et al., 2020; Kothari et al., 2020). Moreover, as consequences of the COVID-19 pandemic, these individuals have been suffering with diminished social interaction with other inmates and outside visitors, suspension of jury trials and delay of court hearings, and recreational and occupational prison activities (Fovet et al., 2020; Hewson et al., 2020; Tozzo et al., 2020). This group is also more vulnerable to the infection of SARS-CoV-2, as prisons tend to be overcrowded, have poorly ventilated environments and low compliance to hygiene rules (Tozzo et al., 2020). In light of this scenario, prisoners are more likely to suffer from anger, depression, anxiety, irritability, frustration, paranoia, fear of contamination, psychosis, exacerbation of underlying mental illness and suicidal behavior (Fovet et al., 2020; Hewson et al., 2020; Tozzo et al., 2020).

### Rural Communities

The individuals living in rural communities experience more loneliness, lack of belonging and perceived burdensomeness than those living in urban centers. They are, therefore, at elevated risk for unsatisfactory mental health and even suicide (Monteith et al., 2020). During the COVID-19 pandemic, the social distancing measures, often not accompanied by virtual connections due to diminished access to the Internet, may exacerbate mental health symptoms and increase suicide behavior in this population (Monteith et al., 2020). Furthermore, intimate partner violence tends to be more intense in rural communities, whereas access to mental healthcare tends to be deficient. All of these circumstances may exacerbate during the pandemic and might result in poorer mental health outcomes and increased suicide rates (Monteith et al., 2020).

### Psychiatric Patients

In psychiatric patients, the COVID-19 pandemic might trigger an even worse outcome regarding mental health. As previously discussed, the uncertainty, fear and social distancing may exacerbate pre-existing psychiatric diseases and precipitate its symptomatology (Holmes et al., 2020; Vindegaard and Benros, 2020; Yao et al., 2020). Added to their higher vulnerability to many stressors (Yao et al., 2020), they face worsen medical follow-up due to the suspension of some elective appointments and redirection of health professionals to face the pandemic (Holmes et al., 2020). Furthermore, they tend to have more severe forms of COVID-19 due to comorbidities, immunosuppression (Fontenelle and Miguel, 2020; Yao et al., 2020) and, possibly, worst access to medical care because of discrimination (Yao et al., 2020).

#### Depression and Anxiety

When performed several scales to assess the psychological impact of COVID-19 pandemic in China on 76 psychiatric patients (with major depressive disorder, anxiety disorders and mixed anxiety and depressive disorder patients) and 109 healthy controls, the patients group had worst outcomes on almost all variables addressing depression, anxiety, stress and insomnia (Hao et al., 2020). As for other psychiatric symptoms referred during the survey, the patients group had more worries about their physical health, more moderate to severe anger and impulsivity and more suicidal ideation (Hao et al., 2020). However, it is important to mention that the control group was evaluated simultaneously to the patients group. Control group was composed of individuals without psychiatric disorders that were evaluated before COVID-19 pandemic. For this reason, results of the study can be contestable.

Moreover, patients with generalized anxiety have increased health anxiety. As a result, they are more prone to confound normal feelings with COVID-19 symptoms, generating even more anxiety and distress (Dubey et al., 2020).

#### Obsessive-Compulsive Disorder

Many patients with obsessive-compulsive disorder (OCD) already excessively worry about having a disease or contaminating others. During this world health emergency, these feelings may intensify (Fontenelle and Miguel, 2020). Also, some signs and symptoms of OCD are very similar to important preventive measures for COVID-19, such as compulsive hand washing and avoiding physical contact (Fontenelle and Miguel, 2020). Therefore, this overlap may cause difficulty for physicians to diagnose and treat new cases of OCD. Finally, the stressors associated with the pandemic might increase the number of new OCD patients, especially among those “at risk” for COVID-19 (Fontenelle and Miguel, 2020).

#### Schizophrenia

In psychiatric patients, excessive attention to media or social networks might precipitate an acute phase of the disease or change its manifestations (Fischer et al., 2020). For example, a 43-year old German patient with schizophrenia had delusions and hallucinations related to the pandemic (Fischer et al., 2020). He believed he contracted the disease through a WhatsApp video from COVID-19 patients in China and started having acoustic hallucinations, anxiety and depressing humor. Therefore, equilibrated communication, based on scientific facts, is essential to minimize this possible damage (Fischer et al., 2020).

Furthermore, schizophrenic patients were less likely to vaccinate, adhere to social distancing, wash their hands and use masks during influenza pandemic (Maguire et al., 2019). This reality is also true for patients with other psychiatric conditions, such as addiction (Narasimha et al., 2020). Therefore, they are a vulnerable group for contracting COVID-19, especially if their mental health is worse than usual (Yao et al., 2020).

#### Hospitalized Patients

Stress experienced during COVID-19 pandemic is probably even higher for psychiatric patients hospitalized for severe illness. In China, these patients had to stay in closed wards without family visits or electronic equipment (Li and Zhang, 2020). These conditions exacerbated their distress and mental symptomatology (Li and Zhang, 2020). Additionally, the patients in these facilities tend to make group activities, share dining and bathroom spaces, interact closely and practice less preventive measures because of their mental state (Bojdani et al., 2020; Xiang et al., 2020). Therefore, they are more vulnerable to the transmission of SARS-CoV-2 (Bojdani et al., 2020; Xiang et al., 2020).

## External Influence

### Culture

As a multi-dimensional psychosocial construct that shapes the perception of the world, culture has the ability to influence several aspects of daily life. In the framework of the pandemic, cultural components affect how the population will perceive, for instance, the implementation of hygienic greetings etiquette, the recognition of health symptoms and the fear of stigmatization (Bruns et al., 2020; Furlong and Finnie, 2020). For this reason, it can be challenging to encourage individuals to comply with some of the necessary precautions, such as avoidance of cultural activities (i.e., worship meetings) and submission to the restricting measures, particularly if these strategies are divergent to the customary social norms (Bruns et al., 2020; Furlong and Finnie, 2020). For example, in Asia, where the COVID-19 outbreak started, discipline is highly valued in society, as well as punishments for deviance. Therefore, the sense of community can be vital in motivating individuals to comply and respect the measures imposed. Contrarily, countries who value freedom and individual expression, including the United States, Italy, and Brazil, may exhibit more difficulties in renouncing personal desires in order to oblige to a common good (Bavel et al., 2020). As a result, the mental health outcomes derived from this pandemic rely deeply on the level of cultural impact in the community (Furlong and Finnie, 2020).

### Media and Access to Information

As a worldwide unprecedented health issue, COVID-19 pandemic has drawn massive attention from the media. Nonetheless, the supply of information regarding the disease has heavily surpassed the demand from the population (Liu and Liu, 2020). This phenomenon has two main repercussions in respect to the mental health impact.

First, the manner by which media vehicles have been portraying the current situation to the community might be causing great harm in terms of psychological implications. This can be easily translated as an analogy with vicarious traumatization, a process suffered by the health staff in command when listening to the victims’ narration of traumatic events they experienced (Liu and Liu, 2020). Similarly, the negative exposure of individuals to ruthless details concerning the COVID-19 pandemic has the potential to promote great psychological distress in the society. During these times, pessimistic reports have prevailed in the correspondence with the public, including daily updates on the number of those infected and number of deaths, economic impact and uncertainty about the future. This consequently increases negative emotions throughout the community and makes people more susceptible to panic. An illustration of this matter is the display of images of empty shelves and panicked shoppers during the first months of the pandemic. Even though this may have been used as a critic, it induced viewers to look out after themselves and foster individuality and competitiveness (Bavel et al., 2020).

Secondly, as information travels at an uncontrollable speed, it is virtually impossible to control the accuracy and authenticity of the majority of news in circulation. As expressed by the Director-General of the WHO, in addition to the fight to the SARS-CoV-2 virus, the world is currently facing an “infodemic” (Dubey et al., 2020; Zarocostas, 2020). It is characterized not only by the massive frequency of fake and inaccurate news (Irwin, 2020), but also conspiracy theories and misinformation, which puts the public through the distress of having to distinguish between scientific evidence and unreliable information (Depoux et al., 2020). The repercussions of this phenomenon are numerous and deleterious, particularly to the mental health of the population, given it is a potential source of anxiety, phobia, panic, depression, obsession, irritability, and COVID-19-related paranoia (Dubey et al., 2020).

### Social Network

In this topic, social networks present twofold effects. On one hand, it is one of the main vehicles of the misinformation and inaccurate information reported. Moreover, people can be adversely influenced by what they see in social networks, which may alter risk perceptions, encourage unhealthy behaviors and reassure the non-compliance with preventative measures (Gao et al., 2020). On the other hand, as the current situation demands physical distancing, remote communication has become an indispensable resource to have social connection (Bergman et al., 2020), as well as to find inspiration on healthy habits and behaviors (Brooks et al., 2020; Gao et al., 2020). In fact, particularly in the scenario of this unprecedented crisis, social networks have been suggested to play a fundamental role in social support, tension release and emotional catharsis (Liu and Liu, 2020).

### Government

In the midst of the pandemic disarray, the government of each country is in the position to guide the population and to execute the necessary interventions to minimize the propagation of the virus. Some strategies consisted of school closures, limited commercial activities, requests that individuals work from home and reduced freedom to use public spaces (Briscese et al., 2020). This kind of leadership has powerful repercussions, with surveys indicating that trust in the government increased from the day the restrictive measures were implemented (Teufel et al., 2020) and that positive behavioral changes were made in response to authorities’ guidance (Atchison et al., 2020). Indeed, it has been demonstrated that government’s interventions of disease prevention and control have significantly increased the likelihood of adoption of protective measures (Duan T. et al., 2020). Moreover, a study showed that, in the United Kingdom, the inclusion of altruism in government’s health messages possibly had a positive effect on wellbeing compared with compulsory orders to stay at home (Holmes et al., 2020).

Nonetheless, in regard to the governments’ strategy to mitigate the emotional and behavioral impact of the pandemic, the results may not be as optimistic. During health crises, there seems to be a special focus from authorities in epidemiological and biomedical data (Atlani-Duault et al., 2020). Indeed, a Chinese study investigated the government’s communication with the public through social media and observed an overall inadequate responsiveness to the public’s concerns. The majority of posts consisted of reports on the epidemic situation, disease-related questions, guidelines and prevention advice. Although these are tremendously relevant topics, there seemed to be insufficient instrumental and emotional support for the community (Liao et al., 2020).

Moreover, an interesting phenomenon is the “heroization” process (Atlani-Duault et al., 2020; Liao et al., 2020). It can be illustrated by the fact that, in any disaster, there seems to be a social need for attribution of blame, in which certain groups or individuals may be considered heroic figures (Liao et al., 2020). Therefore, in order to balance the complex geographies of hope and blame (Atlani-Duault et al., 2020), the government must be aware of this phenomenon in order to modulate the community’s emotional response to the pandemic, as well as to counteract fake news and misinformation (Atlani-Duault et al., 2020).

Another key component to examine in the government’s influence over the population is the establishment of deadlines to the end or the loosening of restricting measures. While the lack of an end date to protective efforts may increase the perceived severity of the situation, which, in turn, may build up compliance to such measures, it is also possible that the absence of a deadline might increase anxiety and other psychological complications over the uncertainty of the future (Briscese et al., 2020). The opposite may also apply: a deadline may also create the impression that the emergency is limited in time and not particularly serious. In addition to that, deadlines may develop expectations in the population that, if not met, might reduce people’s acceptance to the necessary procedures, trust in authorities and compliance to social distancing, a result which is called “social isolation fatigue” (Briscese et al., 2020).

## Measures Adopted to Minimize Behavioral and Psychological Conditions Facing COVID-19 Pandemic

In light of all the harmful ramifications that derive from the current COVID-19 pandemic, it is essential that the government, the health authorities and the population articulate to endorse preventive and supportive measures, not only for the transmission of the disease, but also for emotional, behavioral and psychological impact. In this context, it is important to include mental health professionals managing the pandemic more broadly (Sani et al., 2020). Their knowledge and experience are crucial to monitor the situation and to coordinate supportive measures in order to prevent an even higher increase in psychological disorders, including panic, OCD, addiction and PTSD (Fiorillo and Gorwood, 2020; Sani et al., 2020). Finally, considering that poor mental health is associated with lower adherence to preventive measures for SARS-CoV-2, improving well-being might even decrease the rates of infection (Adhanom Ghebreyesus, 2020).

### Telepsychology Services

As mentioned, worse medical follow-up is one of the most important features to understand the mental health impact of COVID-19 pandemic to mentally ill patients. Demand for telepsychology services, therefore, has increased markedly during the pandemic, and managers have accordingly tried to keep up with this abnormally high demand (Perrin et al., 2020). In fact, psychologists performed 7.07% of their work through telecommunication technologies before the COVID-19 pandemic, whereas, during the pandemic, this number increased to 85.53%, with 67.32% of the mental health professionals executing their work solely via telepsychology (Pierce et al., 2020). The United States government has pursued some actions to enlarge the role of telepsychology, such as allowing the possibility of some drugs to be prescribed in appointments via the Internet, and the expansion of Medicare and Medicaid to cover telepsychology and telemedicine consultations (Perrin et al., 2020; Pierce et al., 2020). Furthermore, online training with experienced trainers, along with tools and resources prepared by psychology organizations, are available in order to instruct the professionals for this new demand (Pierce et al., 2020). These learning opportunities are remarkably important, since lack of self-efficacy is one of the main reasons that explain the small use of telepsychology preceding the pandemic (Pierce et al., 2020).

Concerning its efficacy, Internet-delivered therapy, such as cognitive behavior therapy (CBT), was not less effective than face-to-face CBT in health anxiety disorders, while resulting in lower treatment costs in previous studies (Axelsson et al., 2020). Therefore, even though the evidence base is still limited, these therapeutic modalities may have an important role for facing mental health issues during COVID-19 pandemic (Bilder et al., 2020; Fegert et al., 2020). Nevertheless, telepsychology uptake in more complex cases with severe symptomatology, such as antisocial personality disorder, behavioral issues and bipolar disorder, had a lower increase during the pandemic, indicating difficulty to treat the conditions via the internet or insufficient specialized training for psychologists. The same pattern was observed with testing and evaluation, which could signal insufficient tests adapted for telepsychology (Pierce et al., 2020). Furthermore, technology issues are among the main barriers to the implementation of telehealth. For instance, the elderly may lack familiarity with the platforms, while those from a lower socioeconomic background or those living in rural areas may deal with deficiency of technological devices or Internet connections (Bilder et al., 2020; Monteith et al., 2020).

Finally, mental health professionals should be prepared to address some particularities of minority groups, such as the LGBTQ+, black and latin communities, with cultural responsiveness (Liu and Modir, 2020; Phillips et al., 2020). For the black community, psychologists can use racial socialization in order to enhance awareness about the reality of racism and to promote coping mechanisms (Liu and Modir, 2020).

### Hotlines

It has been recommended that people under quarantine should have access to hotlines with trained healthcare providers to receive guidance regarding possible symptoms or doubts. Such communication channels would reassure and comfort distressed people, providing a sensation that they have not been forgotten. Online support groups for people who are quarantined at home might also be helpful in reducing levels of fear and anxiety (Brooks et al., 2020).

People with suicidal tendencies or previous mental illnesses need special support. Some of them will search for help, and, in this case, it is necessary to increase the volunteer team and to prepare individuals to deal with the situation, including resources through the Internet or phone call. For those who will not search for help, it is important to be attentive to their signs, such as social disconnection and humor flows (Gunnell et al., 2020). Monitoring the mental status of the population is crucial to guide new interventions and improve the overall wellbeing (Holmes et al., 2020).

### Digital Technology Monitoring and Assistance

The global issue of misinformation and social media panic is a crucial topic to be addressed during this pandemic. In order to avoid misleading media reports, the main news outlets should assign professionals to moderate the information that is passed on to the public, in the interest of verifying if it is in line with the current guidelines and scientific evidence (Gunnell et al., 2020). Furthermore, news broadcasts should use simple language when communicating with the public, which means avoiding complex and scientific terms. It is also recommended that they offer practical and specific advice instead of vague or complex guidance (Lakhani et al., 2020; Mesa Vieira et al., 2020). This facilitates the comprehension of the information and also promotes social inclusion of the more vulnerable groups (Mesa Vieira et al., 2020).

Moreover, there is a serious need for online platforms that clarify the available data concerning COVID-19, as well as demystify the fake and inaccurate news in circulation (Depoux et al., 2020). An illustration of this strategy is the “Mythbusters” dashboard in the WHO website, which carries this exact purpose (World Health Organization [WHO], 2020a). Additionally, the use of social media must be conducted with responsibility. The users should avoid the sharing of information that it is either not followed by a reliable source or that may cause panic or anxiety. Medical advice ought to be provided solely if it is backed by evidence (González-Padilla and Tortolero-Blanco, 2020).

Moreover, as the Internet has become an indispensable tool to foster social connections and perform numerous activities, online access, as well as reliable connection, is imperative and must be provided particularly for the vulnerable groups (Bavel et al., 2020). For instance, in order to nurture belonging, webinars, meetings and virtual extracurricular activities can be implemented for the black, latin and LGBTQ+ communities (Liu and Modir, 2020; Salerno et al., 2020). Furthermore, those with limited literacy concerning the use of digital technologies ought to receive special consideration.

### Financial Support

As for the financial stress experienced during the COVID-19 pandemic, the government of each country should offer financial support for the vulnerable population in this context, including the self-employed and those with lower income. It is also crucial to prepare an economical plan during and after the quarantine, in order to reduce stress about the uncertainty of the future (Gunnell et al., 2020). Moreover, aiming to alleviate the economic burden faced particularly by numerous groups throughout the community, medical expenses of COVID-19 confirmed and suspected patients should be subsidized by the government. This strategy may also ensure that individuals seek medical care and, therefore, promotes health equity and disease control amongst the more vulnerable groups (Wang and Tang, 2020).

### Personal Strategies to Improve Mental Health

In light of this difficult scenario, there are ways in which individuals may personally attempt to improve their well-being. Undoubtedly, maintaining interest and motivation is difficult for those suffering from mental health disorders or for those struggling financially (Mental health Covid-19, 2020). Nonetheless, studies have suggested that nourishing adaptive mindsets regarding stress may exert positive effects on how people deal with their emotions. It may also reduce adverse physical symptoms and boost physiological functioning under acute stress (Bavel et al., 2020). In fact, stress and loss of life satisfaction have been associated with higher levels of inflammation, which increases the odds of contracting the disease (Mesa Vieira et al., 2020).

Moreover, a study with employed students observed that there are multiple emotion regulation strategies that might be helpful during this period. They include seeking and reaching out to social connection, such as friends or family, or even volunteering, as reducing the feeling of loneliness and enhancing belongingness is crucial to prevent suicide (Holmes et al., 2020). Keeping oneself committed to other things (i.e., hobbies, music, reading, film, and television and home improvements) and engaging in enjoyable activities to improve one’s mood have also been suggested (Restubog et al., 2020). As complex and multicomponent activities, arts and crafts have been highly associated with diminished risk of developing mental health disorders (Conejero et al., 2020). It is suspected that they modulate several neurotransmitters, as well as cortisol levels, and stimulate neuroplasticity. Therefore, they offer the possibility of emotional expression and regulation (Conejero et al., 2020).

As the COVID-19 outbreak severely restricted people’s movement, outdoor activities have been limited, which does not mean, however, that physical activity needs to be limited as well. Physical exercises have been strongly associated with positive effects regarding mental and physical health (Jiménez-Pavón et al., 2020; Lyons et al., 2020). Therefore, exercising at home is an accessible and easy alternative, which includes not only walking and running, but also several online and free classes of different sport modalities (Chen et al., 2020; Jiménez-Pavón et al., 2020; Mental health Covid-19, 2020).

Several other strategies can contribute to improve the mental health status during the current situation. They include mediation, faith, prayers, playing and listening to music, cooking and baking, caring for a pet or gardening (Lades et al., 2020; Lyons et al., 2020; Mental health Covid-19, 2020). The importance of maintaining a routine or daily plan has also been emphasized (Lyons et al., 2020; Mental health Covid-19, 2020). The management of information intake, keeping news monitoring to a minimum in order to reduce levels of anxiety (Holmes et al., 2020; Mesa Vieira et al., 2020) or simply following official guidelines to stay safe and to respect social distancing are also fundamental strategies to diminish the stress response (Mental health Covid-19, 2020). Furthermore, sleep has a significant impact in mental health and stress response and the population should be constantly informed of its importance, particularly during the COVID-19 pandemic (Holmes et al., 2020).

### Specific Measures for Certain Vulnerable Groups

#### Healthcare Professionals

Considering the emotional and psychiatric risk that healthcare workers are exposed to, actions must also be taken to protect and support this group. Healthcare managers should offer proactive steps to help their workers deal with this situation, reinforcing teams as needed, being honest about the situation and monitoring their staff more closely (Greenberg et al., 2020). Support measures such as psychologists and psychiatrists’ appointments, psychological assistance hotline, support groups and reading materials illustrating coping mechanisms to deal with stressors should also be provided without stigma (Santarone et al., 2020; Zaka et al., 2020). Since many professionals are afraid of going home and infecting their families, it is important to inform them about the safety measures that can minimize the chances of infection (Mamun et al., 2020c). Hospitals can also provide a place where the workers can rest and, if possible, record their hospital routine in order to share with their family (Zaka et al., 2020). Furthermore, the family members of the healthcare professionals should receive special access to testing and treatment, if necessary (Dutheil et al., 2020; Mamun et al., 2020c).

Moreover, an adequate work environment is essential to diminish the mental health impact of the pandemic in healthcare professionals. In that matter, there should be sufficient PPE availability and detailed rules about its use, limited hours in each shift, dissemination of medical information through multiple platforms and languages, education about skills to deal with the patients psychological concerns, delaying of elective appointments and surgeries and, if possible, assembly of a backup force composed of capable retired workers and college students about to graduate for the times of higher patient volume (Dutheil et al., 2020; Santarone et al., 2020; Zaka et al., 2020). Special attention must be paid to preventive strategies of PTSD and its related risk of suicide in the upcoming months (Dutheil et al., 2020).

Finally, it is important to acknowledge that targeting the psychological impact of the pandemic in healthcare professionals is also important to control the COVID-19 itself, since impaired mental health affects their attention, understanding and decision-making (Zaka et al., 2020).

#### Elderly

Considering the particularly detrimental consequences of social disconnection among the elderly, the benefits and damage of such restriction must be thoroughly and continuously weighed (Plagg et al., 2020). In institutions and nursing homes, this group should be allowed the visit of healthy relatives and friends, as long as the hygienic measures are adequately taken. If possible, these relatives and friends might be tested for SARS-CoV-2 infection (Mesa Vieira et al., 2020; Plagg et al., 2020).

Moreover, the family should be encouraged to contact these individuals more frequently, as well as voluntary organizations and community projects should provide similar support for this group (Armitage and Nellums, 2020). Indeed, the phone call outreach program promoted by the Northwestern University in Chicago, Seniors Overcoming Social Isolation, has been developed with the purpose of minimizing social disconnection among the elderly and providing significant engagement with the community (Office et al., 2020). Older adults must also be stimulated to leave their room or even perform outdoor activities when possible (Plagg et al., 2020). Online cognitive behavioral exercises and therapy should also be provided (Armitage and Nellums, 2020).

At last, the community should be encouraged to not comply with ageist content and to recognize the immense value and contribution of the senior citizens to the society (Jimenez-Sotomayor et al., 2020). Furthermore, intergenerational contact ought to be stimulated, as it promotes mental health benefits for both the parties involved (Jimenez-Sotomayor et al., 2020; Office et al., 2020).

#### Children

It has been recommended that the children maintain a healthy routine with adequate sleep cycle and physical activity, and videos can be used to encourage them to exercise and to play (Deslandes and Coutinho, 2020; Wang G. et al., 2020). In order to prevent loneliness, families might seize the opportunity to establish better bonds with their kids, providing them a sense of belonging in the family (Loades et al., 2020). Additionally, social networks should be used to allow interaction with their peers (Loades et al., 2020). Nevertheless, it is suggested that the parents monitor and control the screen time and the content visualized in the Internet (Deslandes and Coutinho, 2020). Parents should always talk to children about the current circumstances clearly and directly, in order to minimize the negative feelings and to help the kids better comprehend the pandemic and the information received from the Internet (Deslandes and Coutinho, 2020; Dubey et al., 2020; Wang C. et al., 2020). Interestingly, an effort that has already been made is the creation of the book *My Hero is You* (Storybook for Children on Covid-19, 2020) by the United Nation in conjunction with other agencies. This book was designed to help children aged 6 to 11 coping with the stress and anxiety generated by the pandemic. Furthermore, online services provided by psychologists can be useful, especially due to domestic conflicts, harassment, abuse and other types of violence (Wang C. et al., 2020).

#### Domestic Violence

Domestic violence is a complex issue with strong cultural components (Gunnell et al., 2020). Therefore, it requires a combination of multiple measures in order to protect the victims (Gulati and Kelly, 2020).

To improve the reporting of domestic violence, it is important to ensure constant availability of hotlines and digital reporting systems (Sacco et al., 2020; Sharma and Borah, 2020). However, since the victims may be isolated with their perpetrators, other alternatives must be adopted. For instance, family, friends and neighbors have an essential role revealing domestic violence, and advertising campaigns should encourage the community to report the cases (Marques et al., 2020; Sacco et al., 2020; Sharma and Borah, 2020; Usher et al., 2020). A positive message, focusing on solutions, is more effective in these circumstances (Sharma and Borah, 2020). Furthermore, code-based systems to report abuse situations could be implemented in pharmacies, supermarkets or even with toll-free phone numbers (Sacco et al., 2020; Usher et al., 2020). Finally, healthcare workers should be aware of the signs of domestic violence and of the risk factors involved, such as substance misuse by family members (Gulati and Kelly, 2020).

After reporting, the speed of the response is critical, especially since the victims and their perpetrators are probably sharing spaces during the pandemic (Sharma and Borah, 2020). Accordingly, domestic violence must be included in the policymaker’s response to the pandemic, guaranteeing financial funding, human resources and protective measures for the victims (Marques et al., 2020; Sharma and Borah, 2020). Moreover, the population should be communicated about the speed of the arrest, since it increases the chances of the victim and bystanders to report the crime (Sharma and Borah, 2020). Finally, encouraging initiatives to provide social support, advocacy and psychological and physical healthcare for the victims is crucial (Gulati and Kelly, 2020; Marques et al., 2020).

Some victims will not report the domestic violence for several reasons, such as fear, economic dependency and protection of the perpetrator. In these cases, friends and family assistance and support groups are especially important to reduce the mental health impact of the abuse (Sharma and Borah, 2020).

#### Informal Settlements and Homeless Individuals

For those living in informal settlements and for the homeless, it is essential that the police avoid top-down forced directives and that committees are created in order to improve communication between the population and the government. Along with financial support, food assistance and adequate water, sanitation and hygiene should be provided for this population (Corburn et al., 2020; Dubey et al., 2020). In this context, non-governmental organizations such as community-based organizations and faith-based groups are also extremely important (Corburn et al., 2020; Dubey et al., 2020). Furthermore, shelters can be created in sports installations, closed universities and military lands to shelter the homeless or to the de-densification of the settlements (Corburn et al., 2020). It is also crucial that psychologists and psychiatrists are available for those demanding specific support (Dubey et al., 2020). Ultimately, all individuals require their basic rights protected to diminish the mental health impact of the pandemic.

#### Prisoners

In light of the increased risk for worse mental health outcomes due to the pandemic, prisoners must receive special consideration and support. Prison management should explore the development of strategies that promote the well-being among the incarcerated community. It has been recommended that the inmates should receive telehealth support, substitute recreational activities, for instance, puzzles, coloring and playing cards, as well as other communication methods, such as writing letters and obtaining increased access to telephone landlines and social networks (Fovet et al., 2020; Hewson et al., 2020; Kothari et al., 2020). They must also be encouraged to practice physical exercise, even if it is inside the cell (Fovet et al., 2020; Hewson et al., 2020). Although access to hygiene measures can be difficult, the prisoners should be constantly informed about the social distancing precautions and provided with accurate information about the pandemic, in order to reduce anxiety and improve adherence to the restricting measures (Hewson et al., 2020; Tozzo et al., 2020).

## Concluding Remarks

COVID-19 pandemic brings novel challenges to human beings. Not only virus’ spread and disease mortality for risk groups, but also emotional, behavioral and psychological impact to the population. Measures to contain disease transmission, including quarantine, social isolation and social distancing may affect the population’s behavior and may lead to psychological disorders. Several emotional and psychological conditions including fear, anxiety, depression, and suicide ideation are triggered by the pandemic itself as well as by the adopted preventive measures. Special attention should be paid to vulnerable groups both in regard to prevent harmful emotional repercussions of the pandemic, but also to provide the necessary assistance. The health authorities and the governments should strategize to alleviate the mental burden of COVID-19 pandemic by providing emotional support to the entire population, but particularly to the vulnerable individuals.

## Author Contributions

ALP and LB equally contributed to this article as first authors, both authors made the literature revision and selection of main articles for this review, defined the topics of this review, and wrote the first draft. ACFF, MLBC, RGBC, and SBCSB helped in writing specific topics of the review and helped in search and analysis of clinical trials included in this review. ACSS conceptualized the study, made general supervision, revised the manuscript, and submitted the final version of the manuscript, which is approved by all authors.

## Conflict of Interest

The authors declare that the research was conducted in the absence of any commercial or financial relationships that could be construed as a potential conflict of interest.
